# Letter from the Editor in Chief

**DOI:** 10.19102/icrm.2025.16051

**Published:** 2025-05-15

**Authors:** Devi Nair


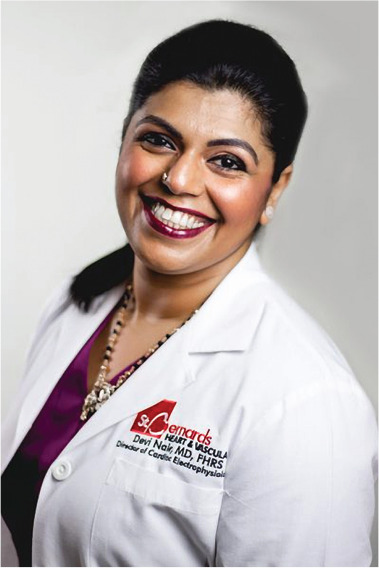


Dear Colleagues,

We have just concluded a truly energizing Heart Rhythm 2025 in San Diego, and I’m proud to share the momentum with you through this May 2025 issue of *The Journal of Innovations in Cardiac Rhythm Management*. The meeting brought together a vibrant community of over 10,000 electrophysiologists, industry innovators, fellows, and patients from around the world. It was a celebration of science, advocacy, and connection—one that clearly demonstrated the strength and unity of our field.

## Heart Rhythm 2025: A Pulse on the Future

### Key Studies

The late-breaking clinical trials this year were among the most impactful to date. We saw:

- Promising outcomes for conduction system pacing in the I-CLAS study, where left bundle branch area pacing (LBBAP) significantly reduced heart failure hospitalizations and mortality compared to traditional biventricular pacing;
- A compelling GLP-1 receptor agonist study showing a substantial reduction in atrial fibrillation (AF) recurrence whose findings strengthen the link between metabolic disease and arrhythmia burden, reinforcing the need for integrated care;
- New data on artificial intelligence–assisted signal processing in AF mapping that may help reduce ablation times while increasing safety—technology that’s already beginning to shape how we think about substrate-guided workflows; and
- Advancements in pulsed field ablation (PFA) technologies, with multiple studies showcasing improved procedural efficiency and safety profiles, including reduced collateral damage to surrounding tissues. In particular, these studies included:
  - The PULSAR investigational device exemption trial evaluating the Globe^®^ pulsed field system (Kardium, Burnaby, BC, Canada), which reported a 78% rate of freedom from AF at 1 year in patients with paroxysmal AF, with over 94% of pulmonary veins successfully isolated on first application and an absence of device-related primary safety events, along with impressive procedural metrics.
  - The Omny-IRE trial, a first-in-human study of the OMNYPULSE™ platform (Biosense Webster, Diamond Bar, CA, USA), which reported an achievement of 100% acute pulmonary vein isolation success, with durable isolation in 84.5% of veins at 3 months. Integration with the CARTO™ 3 system (J&J MedTech, New Brunswick, NJ, USA) and very low adverse event rates make this technology a compelling addition to the growing PFA landscape.
  - The VCAS trial, which focused on scar-related ventricular tachycardia (VT), demonstrated that PFA may provide transmural lesion formation and 78% freedom from VT, even in patients with dense fibrotic tissue. This represents a potentially groundbreaking application of PFA in the VT space.
  - Other key PFA sessions included insights from the NEMESIS PFA trial, which examined the potential off-target effects and complications associated with PFA, contributing to our evolving understanding of safety.

These findings suggest that we are rapidly moving toward next-generation ablation strategies that are faster, safer, and more effective.

### PFA Live Case Summit 2025

Complementing Heart Rhythm 2025 was the PFA Live Case Summit 2025, held April 24. This one-day global broadcast featured 15 live and 8 recorded cases from leading electrophysiology centers across the United States, Canada, and Europe. Moderated by a global faculty of 66 key opinion leaders, the summit offered an unparalleled look at PFA’s diversity, spanning various platforms and approaches. With a format that wove in slides of high-impact publications during live procedures, it supplied a masterclass in both technique and critical thinking for contemporary PFA adoption. The summit provided attendees with firsthand insights into the application of PFA in clinical settings, emphasizing its potential to enhance procedural safety and efficacy. Key opinion leaders engaged in in-depth discussions, sharing their experiences and perspectives on the integration of PFA into contemporary electrophysiological practice.

### Exhibit Hall Programming

The Rhythm Theaters were again a major draw—providing a space for live demonstrations, new product showcases, and hands-on learning with experts. The Exhibit Hall buzzed with innovation as companies shared their latest advances in ablation, imaging, pacing, and digital health. That spirit of curiosity and collaboration was felt in every corner.

### Advocacy and Voice: The Role of Our Community

Equally moving was the Heart Rhythm Advocacy Gala, which reinforced the importance of having a collective voice as the electrophysiology community. Whether it’s advancing health equity, expanding access to lifesaving therapies, or shaping health policy, advocacy is central to the future of our field. This year’s gala was not only a celebration of leadership and service but also a call to action: We must remain present—not just in the lab and clinic but also in the public discourse that defines patient care access and standards.

### Leadership in Transition

We also marked an important leadership transition within the Heart Rhythm Society. We extend our deepest gratitude to Dr. Kenneth A. Ellenbogen for his steadfast leadership over the past year, and we warmly welcome Dr. Mina K. Chung as HRS President for 2025–2026. Her vision, scientific rigor, and collaborative energy will continue to elevate our specialty.

## Featured Articles in This Issue

This month’s issue reflects the clinical themes presented at HRS, with three timely contributions:

- **“Learning Curve for Left Bundle Branch Area Pacing Lead Implantation” by Clark et al.** This multicenter study shows how growing operator experience with LBBAP translates to reduced fluoroscopy times and higher implant efficiency—fitting in the context of late-breaking evidence presented this year.
- **“Right-sided Cardiac Resynchronization Therapy via LBBAP in a Patient with Persistent Left Superior Vena Cava” by Menemencioglu and Canpolat.** This is a compelling case that demonstrates the flexibility of conduction system pacing even in patients with unusual anatomy, underscoring the technique’s expanding utility.
- **“Zero-fluoroscopy Pulsed-field Ablation for AF” by Harmouch et al.** This technically impressive case used four-dimensional intracardiac echo to guide PFA entirely without fluoroscopy—timely as the community continues to prioritize radiation safety and efficiency.

As we move forward from San Diego and back to our practices and labs, let’s carry with us the innovation, collegiality, and purpose that define Heart Rhythm Society’s annual gathering. The spirit of Heart Rhythm 2025 reminds us that our progress is not only technical but also personal. Through scientific discovery, clinical creativity, and unwavering collaboration, we shape a field that is as dynamic as it is human. Thank you to our contributors, readers, and the entire electrophysiology community for your continued engagement with the journal. I look forward to the insights and energy we’ll carry from San Diego into the next chapter of innovation.

Warm regards,


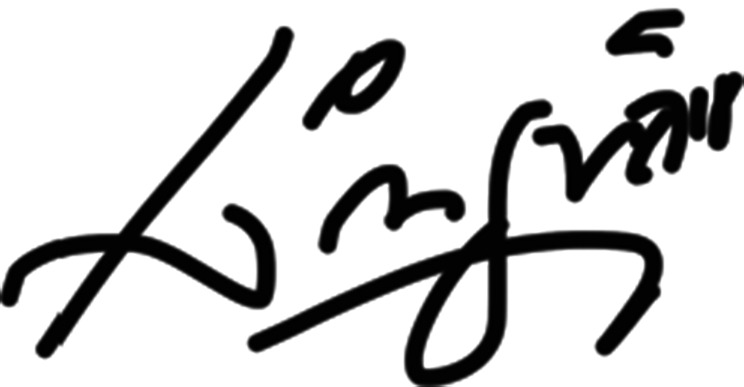


Dr. Devi Nair, md, facc, fhrs

Editor-in-Chief

*The Journal of Innovations in Cardiac Rhythm Management*

Director of the Cardiac Electrophysiology & Research,

St. Bernard’s Heart & Vascular Center, Jonesboro, AR, USA

White River Medical Center, Batesville, AR, USA

President/CEO, Arrhythmia Research Group

Clinical Adjunct Professor, University of Arkansas for Medical Sciences

Governor, Arkansas Chapter of American College of Cardiology

drdgnair@gmail.com

